# Comparison of the efficacy of different androgens measured by LC-MS/MS in representing hyperandrogenemia and an evaluation of adrenal-origin androgens with a dexamethasone suppression test in patients with PCOS

**DOI:** 10.1186/s13048-021-00781-5

**Published:** 2021-02-14

**Authors:** Fu Chen, Minjie Chen, Weichun Zhang, Huihuang Yin, Guishan Chen, Qingxia Huang, Xiaoping Yang, Lan Chen, Chujia Lin, Guoshu Yin

**Affiliations:** 1 Department of Clinical Nutrition, The 1st Affiliated Hospital of Shantou University Medical College, 57 Changping Road, Shantou, 515041 Guangdong China; 2 Department of Endocrinology, The 1st Affiliated Hospital of Shantou University Medical College, 57 Changping Road, Shantou, 515041 Guangdong China; 3 Laboratory of Molecular Cardiology and Laboratory of Molecular Imaging, The 1st Affiliated Hospital of Shantou University Medical College, 57 Changping Road, Shantou, 515041 Guangdong China

**Keywords:** Polycystic ovary syndrome (PCOS), Dexamethasone suppression test, Androgen, Adrenal, Liquid chromatography-mass spectrometry (LC-MS/MS)

## Abstract

**Background:**

The aims of this study were to compare the efficacy of different androgens measured by liquid chromatography-mass spectrometry (LC-MS/MS) in representing hyperandrogenemia and to evaluate adrenal-origin androgens with a dexamethasone suppression test in patients with polycystic ovary syndrome (PCOS).

**Methods:**

One hundred and two patients with PCOS and 41 healthy volunteers were recruited and total serum testosterone (TT), androstenedione (AD), dehydroepiandrosterone (DHEA) and dehydroepiandrosterone sulfate (DHEA-S) were measured by LC-MS/MS. ROC analysis was performed to compare the efficacy of different androgens in representing hyperandrogenemia. Dexamethasone suppression test was performed in 51 patients with PCOS and above indicators were measured after dexamethasone administration. The prediction efficacy of DHEA and DHEA-S at baseline in the dexamethasone suppression test was evaluated with ROC analysis.

**Results:**

The AUCs of TT, AD, free androgen index (FAI) and DHEA-S in ROC analysis for representing hyperandrogenemia were 0.816, 0.842, 0.937 and 0.678, respectively. The optimal cutoff value of TT was 0.337 ng/ml, with a sensitivity of 72.0% and specificity of 82.93%. The optimal cutoff value for AD was 1.309 ng/ml, with a sensitivity of 81.0% and specificity of 73.17%. The optimal cutoff value of the FAI was 2.50, with a sensitivity of 87.0% and specificity of 92.68%. Alternatively, AD or FAI more than the optimal cutoff values as evidence of hyperandrogenemia had the highest sensitivity of 91.18%. The levels of cortisol, DHEA and DHEA-S were all suppressed to narrow ranges after dexamethasone administration. Nine and 8 of 51 patients with PCOS had significant decreases in TT and AD, respectively. DHEA can be used as a indicator for predicting significant decrease of TT in dexamethasone suppression test with cutoff value of 13.28 ng/ml**.** A total of 27.5% (14/51) of patients had DHEA-S excess, but only 1 of 9 patients who had a significant decrease in TT had elevated level of DHEA-S at baseline.

**Conclusions:**

AD measured by LC-MS/MS can represent hyperandrogenemia in PCOS patients and, combined with TT or FAI, can improve the screening efficiency of hyperandrogenemia. Seventeen percent of PCOS patients had adrenal-origin androgen dominance, with TT significantly decreasing after 2 days of dexamethasone administration. Adrenal-origin androgen dominance was not parallel with DHEA-S excess in patients with PCOS.

## Introduction

Polycystic ovary syndrome (PCOS), with a prevalence ranging from 6 to 20%, is the most common endocrine disorder that causes infertility in reproductive-aged women, and it is characterized by hyperandrogenemia, chronic anovulation, and polycystic ovary morphology [1, 2]. Androgen excess is one of the main features and diagnosis points of PCOS. Serum total testosterone (TT) remains the most commonly determined and widely used marker of biochemical androgen excess in our routine clinical work. O’Reilly and his coworkers reported that androstenedione (AD) can be increased when TT is normal in some patients with PCOS and suggested that AD can be a sensitive indicator for androgen excess [3]. The methods of determination of androgen and its precursors, such as direct immunoassays, affect the accuracy of the results. Androgen and its precursors are recommended to be measured by liquid chromatography-mass spectrometry (LC-MS/MS) [3, 4], which can overcome the disadvantages of direct immunoassays.

The adrenals and ovaries share similar basic steroidogenic enzymatic machinery, and it is difficult to identify androgen from different origins by blood biochemical tests. However, ovarian androgen synthesis is regulated by luteinizing hormone (LH), whereas adrenal androgen synthesis is regulated by adrenocorticotropin (ACTH). Dexamethasone administration can suppress androgen secretion originating from the adrenal gland, and a significant decrease in androgen may represent circulating androgen dominantly originating from the adrenal gland. Dehydroepiandrosterone sulfate (DHEA-S), after peripheral conversion to dehydroepiandrosterone (DHEA), is further converted to AD and contributes indirectly to increased extraovarian testosterone formation [5]. DHEA-S was used as an index for adrenal androgen excess evaluation. Whether adrenal androgen excess and androgen dominantly originating from the adrenal gland are parallel is still unknown.

The aims of this study were to compare the efficacy of different androgens measured by LC-MS/MS in representing hyperandrogenemia and to evaluate adrenal-originated androgens with a dexamethasone suppression test in patients with PCOS.

## Subjects and methods

### Subjects

The study was approved by the Ethics Committee of the First Affiliated Hospital Shantou University Medical College according to the Council for International Organizations of Medical Sciences. All participants were recruited from the Department of Endocrinology at the First Affiliated Hospital Shantou University Medical College between September 2018 and July 2019. Written informed consent was obtained from all participants. Women with PCOS were diagnosed according to the 2003 Rotterdam criteria, which require the presence of at least two of the following: (1) oligo-ovulation and/or anovulation; (2) clinical and/or biochemical signs of hyperandrogenism; and (3) ultrasound findings of polycystic ovaries in 1 or 2 ovaries, ≥12 follicles measuring 2 to 9 mm in diameter, and/or ovarian volume ≥ 10 ml. Diagnoses of PCOS were made after the exclusion of other etiologies for hyperandrogenemia or ovulatory dysfunction (Cushing syndrome, 21-hydroxylase deficiency, thyroid disease, androgen-secreting tumors, congenital adrenal hyperplasia and hyperprolactinemia) [6]. All individuals with PCOS were first-visit patients and had not received PCOS-related treatment.

The healthy volunteers were recruited via local advertisement from the general community. These healthy volunteers had regular menstrual cycles and normal ovarian morphology. Regular menstrual cycle was defined as menstrual flow occur every 21 to 35 days and last 2 to 7 days. Transvaginal or transrectal ultrasonography was used to assess uterine and ovarian morphology on day 2–5 days of the menstrual cycle and individuals with polycystic ovarian changes were excluded. Individuals who had clinical features of androgen excess, and who were breastfeeding or pregnant within the past year were also excluded from the study. In order to exclude the possible effect of abnormal metabolism on androgens in control population, 41 healthy volunteers with normal blood lipid, blood glucose and BMI were included in this study.

### Procedure

All the participants were asked to come to our department during days 2–4 of spontaneous cycles after an overnight fast. Height, body weight and body mass index (kg/m^2^) were calculated. Blood pressure was measured after at least 15 min of rest. Peripheral blood samples were collected from all subjects for TT, AD, DHEA and DHEA-S, sex hormone binding globulin (SHBG), LH, and follicle-stimulating hormone (FSH) measurement.

A 2-day suppression test with 2 mg of dexamethasone per day was performed in 51 patients with PCOS. Serum TT, AD, DHEA, DHEA-S and cortisol were measured after 2 days of dexamethasone administration (0.5 mg every 6 h for 48 h). If there was a significant decrease (below 50% of the baseline value) in serum TT following the 2-day dexamethasone administration, then the diagnosis of hyperandrogenism of adrenal-origin dominance can be made [7].

### Assays

A liquid chromatography (ACQUITY UPLC I Class, Water, MA, USA) coupled to tandem mass spectrometry (Triple Quad™5500, AB SCIEX, MA, USA) system were used to quantitate TT, AD, DHEA and DHEA-S with the multiple reaction monitoring (MRM) model in BGI (Shenzhen, China). Steroid hormone test kit was provided by BGI·GBI (Beijing, China) and the internal standards included testosterone-IS, A4-IS, HDEA-IS and DHEA-S-IS. Calibrators (*n* = 7) were prepared with testosterone concentrations ranging from 0.01 ng/ml to 10 ng/mL, with AD concentrations ranging from 0.008 ng/ml to 10 ng/ml, with DHEA concentrations ranging from 0.03 ng/ml to 30 ng/ml and with DHEA-S concentrations ranging from 4.8 ng/ml to 4800 ng/ml, respectively. Measurement preparation began with 250 μl of sample added to glass tubes followed by the addition of 200 μl of the internal standard mixture and well vortexed. Activated SPE plate (Oasis®HLB 96-Well Plate 30 μm, Water, MA, USA) with 1.0 ml dichloromethane, 1.0 ml acetonitrile and 1.0 ml methanol in turn, and then added 1.0 ml ultra pure water for balancing twice. All the pretreated samples were transferred to the homogenized SPE plate and passed through the column and the waste liquid was discarded. Then 500.0 μl 25% methanol were added and passed through the column. Discarded the waste liquid, and repeated once, and then completely dried the SPE column. Added 1.20 ml dichloromethane to the column to elute steroid hormones and the elute was under a nitrogen airstream. After drying, 60.0 μl 25% methanol was added into the receiving plate, vortexed for 5 min, and then centrifugated (2000 g, 4 °C, 5 min). Transferred 50.0 μl of supernatant to the sample plate and prepared for measurement. Separation was performed on a Kinetex® C18 column (2.1 × 50 mm, 2.6 μm) (Phenomenex, CA, USA) which was maintained at 55 °C. The mobile phase, consisting of 1 mM ammonium acetate in water (solvent A) and 1 mM ammonium acetate in methanol (solvent B), was delivered at a flow rate of 0.80 ml/min. The solvent gradient was set as follows: initial 25% B; 40% B, 1.8 min; 70% B, 3.8 min; 95% B, 3.9 min; 95% B, 5.5 min; 25% B, 5.51 min; 25% B, 6.5 min. The mass spectrometer was operated in positive MRM mode. Parameters were as follows: IonSpray Voltage (5500 V), Curtain Gas (40 psi), Ion Source Temp (650 °C), Ion Source Gas of 1 and 2 (60 psi).

SHBG was measured using the luminescence immunoassay (Siemens, New York, USA) with intra- and total CVs of < 5.3 and < 6.6%, respectively. Levels of serum FSH, LH and cortisol were measured by radioimmunoassay (Beckman Coulter, CA, USA). The free androgen index (FAI) was calculated with the formula FAI = TT(ng/ml)*100*3.467/SHBG (nmol/L) (1 ng/ml = 3.467 nmol/L for TT).

### Statistical analysis

The area under the curve (AUC) for the receiver operating characteristic (ROC) curves, the cutoff values, and the test performance characteristics were obtained from MedCalc. The optimal ratio value for each clinical condition was defined as the value on the ROC curve that was associated with the minimum Euclidean distance from the curve to the upper left corner of the graph using Youden’s index according to the formula $\sqrt{{\left(1- sensitivity\right)}^2+{\left(1- specificty\right)}^2}$. Other statistical analyses were performed with SPSS 22.0. The data are expressed as the mean ± the SD. A t-test was performed to determine differences between groups. Pearson correlation analysis was used to determine the relationship between variables. Multiple linear regression was used to screen factors influencing TT in patients with PCOS and healthy volunteers. A value of *P* < 0.05 was considered statistically significant.

## Results

### Clinical and hormonal variables in patients with PCOS and healthy volunteers

In this study, 102 patients with PCOS and 41 healthy volunteers were recruited. Clinical and hormonal variables are described in Table 1. PCOS patients had higher levels of BMI (*P* < 0.001), TT (P < 0.001), AD (P < 0.001), DHEA-S (*P* = 0.002), FAI (P < 0.001), LH (P < 0.001) and FSH (*P* = 0.039) and lower levels of SHBG (P < 0.001) than the control group. Age and DHEA were not significantly different between these two groups.

**Table 1:** Clinical and hormonal characteristics in PCOS patients and healthy volunteers

Variables	PCOS	Healthy volunteers	***P*** value
**n**	102	41	–
**Age (years)**	**29.02 ± 3.75**	29.39 ± 4.23	0.628
**BMI (kg/m2)**	24.32 ± 4.52	20.30 ± 1.77	< 0.001
**TT (ng/ml)**	0.50 ± 0.23	0.27 ± 0.12	< 0.001
**AD (ng/ml)**	1.94 ± 0.75	1.15 ± 0.37	< 0.001
**DHEA (ng/ml)**	10.52 ± 6.42	10.05 ± 5.10	0.680
**DHEA-S (ng/ml)**	2948.79 ± 1059.11	2340.28 ± 951.74	0.002
**LH (mIU/ml)**	10.64 ± 6.17	4.23 ± 1.81	< 0.001
**FSH (mIU/ml)**	6.82 ± 2.01	6.09 ± 1.52	0.039
**SHBG (nmol/L)**	35.61 ± 22.33	66.70 ± 26.35	< 0.001
**FAI**	6.49 ± 4.63	1.57 ± 0.85	< 0.001

### Correlations of different kinds of androgen and screening for the factors influencing TT

The results of the correlation analysis among different kinds of androgen in PCOS patients and healthy volunteers are shown in Table 2. For the PCOS patients, TT was positively correlated with AD (r = 0.799, *P* < 0.001) and DHEA (r = 0.382, *P* < 0.001) but not with DHEA-S. AD was positively correlated with DHEA (r = 0.447, P < 0.001) and DHEA-S (r = 0.201, *P* = 0.043). DHEA was positively correlated with DHEA-S (r = 0.321, *P* < 0.001). For the healthy volunteers, TT was positively correlated with AD (r = 0.821, P < 0.001) but not with DHEA and DHEA-S. AD was positively correlated with DHEA (r = 0.647, P < 0.001) and DHEA-S (r = 0.400, *P* = 0.009). DHEA was positively correlated with DHEA-S (r = 0.459, *P* = 0.003). All the subjects were taken into consideration, and TT, AD, DHEA and DHEA-S all had positive correlations with each other.

**Table 2:** Correlations of different androgens in PCOS patients and healthy volunteers

Correlations of variables	PCOS			Healthy volunteers			Total	
	r	P value		r	P value		r	P value
**TT and AD**	0.799	< 0.001		0.821	< 0.001		0.843	< 0.001
**TT and DHEA**	0.382	< 0.001		0.250	0.115		0.337	< 0.001
**TT and DHEA-S**	0.102	0.309		0.180	0.260		0.213	0.011
**AD and DHEA**	0.447	< 0.001		0.647	< 0.001		0.427	< 0.001
**AD and DHEA-S**	0.201	0.043		0.400	0.009		0.315	< 0.001
**DHEA and DHEA-S**	0.321	0.001		0.459	0.003		0.348	< 0.001

TT was most commonly used for PCOS diagnosis according to the instructions of the PCOS guidelines [8]. To screen the factors influencing TT, multivariate linear regression analysis was conducted, and the results are shown in Table 3. SHBG (β = 0.311, *P* < 0.001), AD (β = 0.834, P < 0.001) and BMI (β = 0.161, *P* = 0.024) were factors influencing TT in PCOS patients, whereas SHBG (β = 0.256, *P* = 0.005) and AD (β = 0.868, P < 0.001) were factors influencing TT in healthy volunteers.

**Table 3:** Multivariate regression analysis of TT in PCOS patients and healthy volunteers

Subjects recruited	Variables	β	P value
**PCOS**	SHBG	0.311	< 0.001
	BMI	0.161	0.024
	AD	0.834	< 0.001
**Healthy volunteers**	SHBG	0.256	0.005
	AD	0.868	< 0.001

### Comparison of the value of different androgens for PCOS screening

**Fig. 1:**
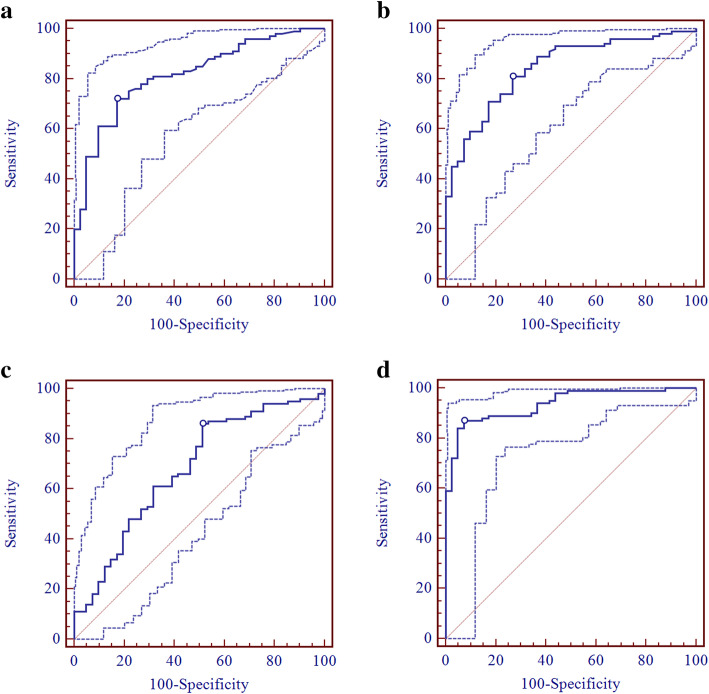
ROC analysis of different androgens for representing hyperandrogenemia in PCOS patients using LC-MS/MS. The AUCs of TT, AD, DHEAS and the FAI in ROC analysis were 0.816, 0.842, 0.678 and 0.937, respectively. **a**: TT; **b**: AD; **c**: DHEA-S; **d**: FAI. The dotted line represents the 95% confidence interval

To compare the value of different androgens for representing hyperandrogenemia in PCOS patients, ROC analysis was performed and the results are shown in Table 4 and Fig. 1. The AUCs of TT, AD, DHEA-S and the FAI in ROC analysis were 0.816 (95% CI: 0.742–0.876, *P* < 0.001), 0.842 (95% CI: 0.771–0.898, P < 0.001), 0.678 (95% CI: 0.594–0.754, P < 0.001) and 0.937 (95% CI: 0.883–0.971, P < 0.001), respectively. The AUC of the FAI was significantly larger than that of AD, TT and DHEA-S (all P < 0.001), whereas the AUCs of AD and TT were significantly larger than that of DHEA-S (*P* < 0.01 and P < 0.001, respectively). The AUCs of TT and AD were not significantly different. DHEA had no value in screening for PCOS.

**Table 4:** AUC and test characteristics of different androgens for representing hyperandrogenemia in PCOS patients using ROC analysis

Androgens	AUC	95% CI	Z value	P value	Optimal cutoff value of androgens	Sensitivity (95% CI)	Specificity (95% CI)	Positive predictive value (95% CI)	Negative predictive value (95% CI)	Positive LR (95% CI)	Negative LR (95% CI)
TT	0.816	0.742–0.876	8.894	< 0.001	0.337	72.0 (62.1–80.5)	82.93 (67.9–92.8)	91.1 (82.6–96.3)	54.8 (41.7–67.5)	4.22 (3.5–5.1)	0.34 (0.2–0.7)
AD	0.842	0.771–0.898	10.489	< 0.001	1.309	81.0 (71.9–88.2)	73.17 (57.1–85.8)	88.0 (79.6–93.9)	61.2 (46.2–74.8)	3.02 (2.5–3.7)	0.26 (0.1–0.3)
DHEA-S	0.678 ^ac^	0.594–0.754	3.792	< 0.001	1887.057	86.0 (77.6–92.1)	48.78 (32.9–64.9)	80.4 (71.6–87.4)	58.8 (40.7–75.3)	1.68 (1.2–2.3)	0.29 (0.2–0.5)
FAI	0.937 ^bcd^	0.883–0.971	22.389	< 0.001	2.50	87.0 (78.8–92.9)	92.68 (80.1–98.4)	96.7 (90.6–99.3)	74.5 (60.4–85.7)	11.89 (10.6–13.3)	0.14 (0.04–0.5)

Compared with AUC_TT_, a: P < 0.01, b: P< 0.001

Compared with AUC_AD_, c: P< 0.001

Compared with AUC_DHEA-S_, d: P< 0.001

The optimal cutoff value of TT was 0.337 ng/ml, with a sensitivity of 72.0% (95% CI: 62.1–80.5%) and specificity of 82.93% (95% CI: 67.9–92.8%). The optimal cutoff value for AD was 1.309 ng/ml, with a sensitivity of 81.0% (95% CI: 71.9–88.2%) and specificity of 73.17% (95% CI: 57.1–85.8%). The optimal cutoff value of DHEA-S was 1887.057 ng/ml, with sensitivity 86.0% (95% CI: 77.6–92.1%) and specificity 48.78% (95% CI: 32.9–64.9%). The optimal cutoff value of the FAI was 2.50, with a sensitivity of 87.0% (95% CI: 78.8–92.9%) and specificity of 92.68% (95% CI: 80.1–98.4%).

Levels of TT and AD higher than their optimal cutoff values were defined as high levels of TT and AD, respectively. Eleven patients with PCOS had elevated levels of AD but normal levels of TT, whereas only 2 patients had elevated levels of TT but normal levels of AD. The other 89 patients had the same trend in TT and AD.

To improve the values of different androgens for representing hyperandrogenemia for patients with PCOS, combined androgens were used for analysis, and the results are shown in Table 5. For AD or TT more than the optimal cutoff values representing hyperandrogenemia, the sensitivity increased to 83.33%, with a specificity of 70.73%. Simultaneously, for AD and TT more than the optimal cutoff values as evidence of hyperandrogenemia, the sensitivity and specificity were 70.59 and 80.49%, respectively. Alternatively, for AD or an FAI more than the optimal cutoff values representing hyperandrogenemia, the sensitivity and specificity were 91.18 and 68.29%, respectively. Simultaneously, for AD and an FAI more than the optimal cutoff values as evidence of hyperandrogenemia, the sensitivity dropped to 73.53%, and the specificity maintained in 92.68%.

**Table 5:** vb characteristics of combined androgens for representing hyperandrogenemia in PCOS patients

Combined androgens	Sensitivity (%)	Specificity (%)	Positive predictive value (%)	Negative predictive value (%)
**Elevated AD or TT**	83.33	70.73	87.63	63.04
**Elevated AD and TT**	70.59	80.49	90.00	52.38
**Elevated AD or FAI**	91.18	68.29	87.74	75.68
**Elevated AD and FAI**	73.53	92.68	96.15	58.46

### Results of the dexamethasone suppression test

**Fig. 2:**
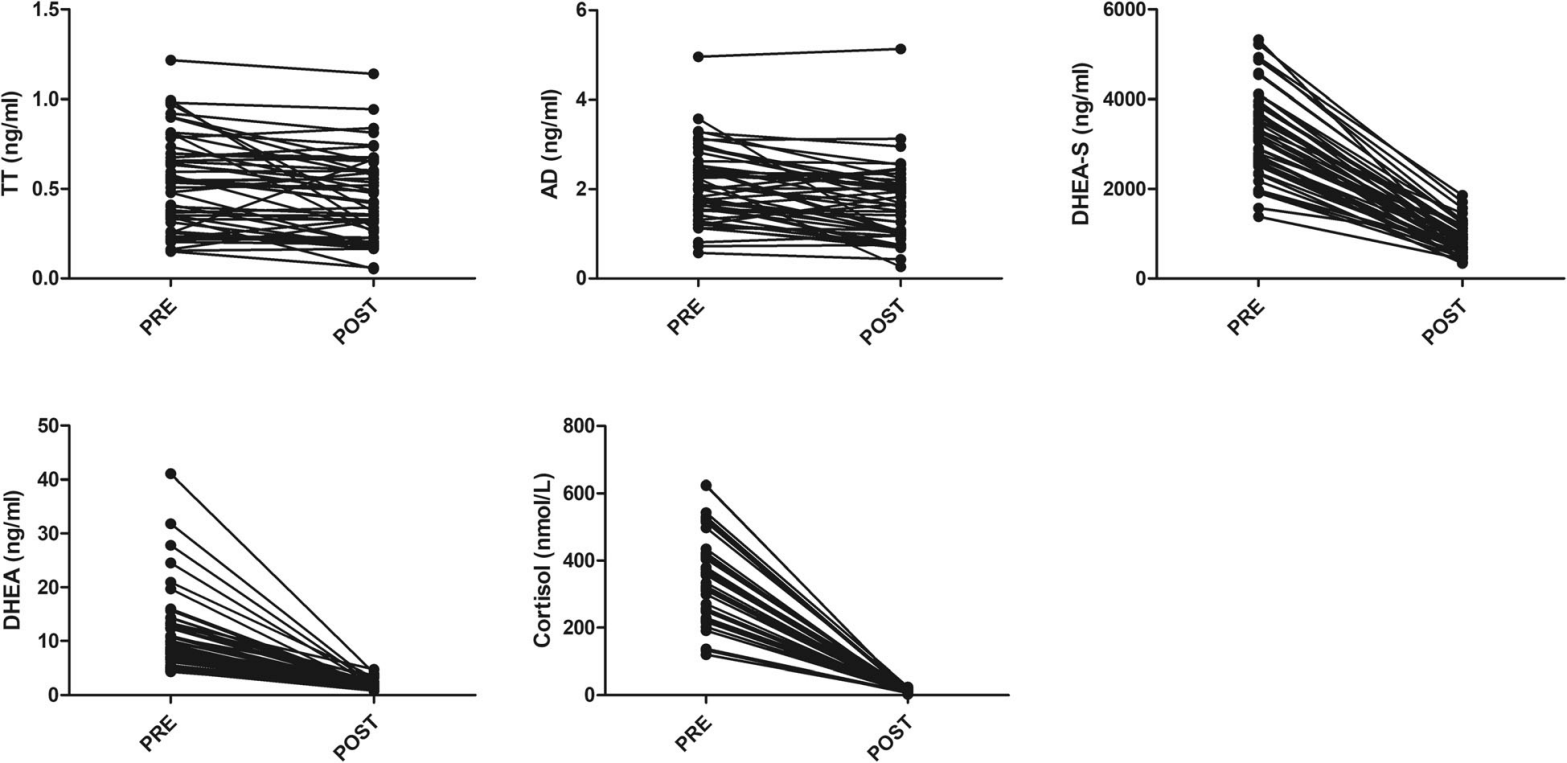
Results of TT, AD, DHEA, DHEA-S and cortisol before and after 2 days of dexamethasone administration. The levels of cortisol, DHEA and DHEA-S were all suppressed to narrow ranges after 2 days of dexamethasone administration, especially for the levels of cortisol and DHEA. The changes in TT and AD were parallel. Nine and 8 of 51 patients with PCOS had significant decreases (below 50% of the baseline value) in TT and AD, respectively, following the 2-day dexamethasone administration. PRE: Levels of androgens before dexamethasone administration; POST: Levels of androgens after dexamethasone administration

**Fig. 3:**
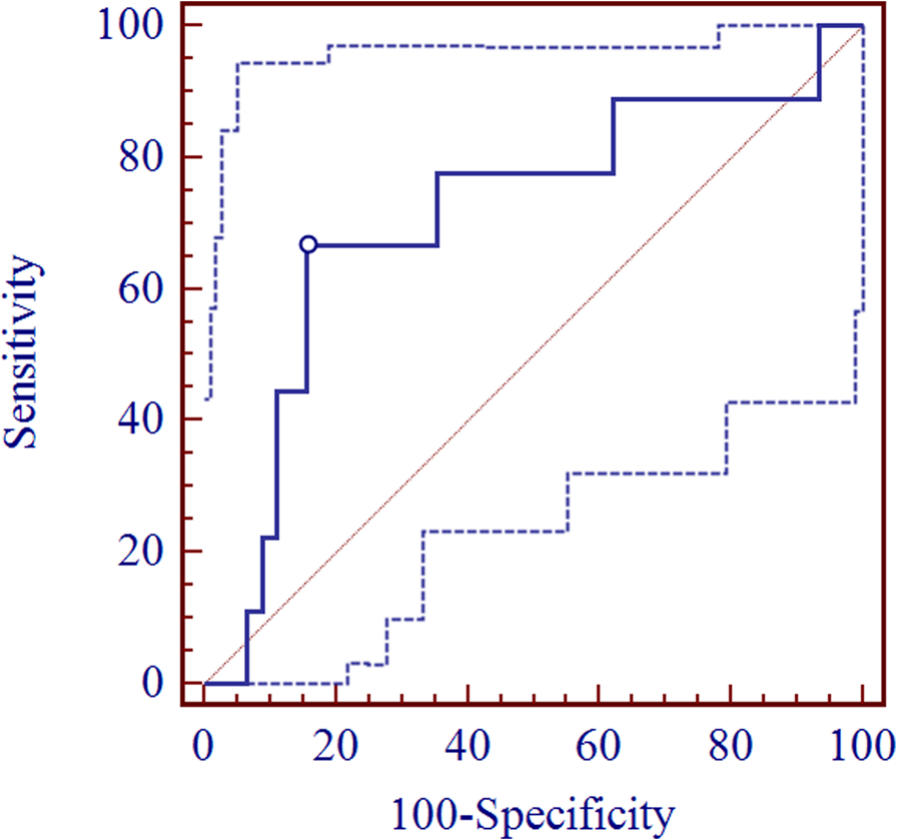
ROC analysis of DHEA for predicting a significant decrease in TT after 2 days of dexamethasone administration. The AUC of the ROC curve was 0.711, and the optimal cutoff value was 13.28 ng/ml, with a sensitivity of 66.67% (30.1–92.1%) and a specificity of 84.44% (70.5–93.5). The dotted line represents the 95% confidence interval

A two-day dexamethasone suppression test was performed in 51 patients with PCOS, and the results are shown in Fig. 2. The levels of cortisol (PRE: 354.76 ± 120.69 nmol/L, POST: 9.87 ± 4.32 nmol/L), DHEA (PRE: 11.29 ± 7.25 ng/ml, POST: 1.87 ± 0.81 ng/ml) and DHEA-S (PRE: 3220.39 ± 998.27 ng/ml, POST: 871.90 ± 378.72 ng/ml) were all suppressed to narrow ranges after 2 days of dexamethasone administration, especially the levels of cortisol and DHEA. After dexamethasone administration, the change of TT (PRE: 0.54 ± 0.26 ng/ml; POST: 0.43 ± 0.24 ng/ml) was consistent with that of AD (PRE: 2.08 ± 0.83 ng/ml; POST: 1.63 ± 0.86 ng/ml). Nine and 8 of 51 patients with PCOS had significant decreases (below 50% of the baseline value) in TT and AD, respectively, following the 2-day dexamethasone administration. To evaluate the predictive value of DHEA at baseline for the dexamethasone suppression test, ROC analysis was performed. Nine patients with a significant decrease in TT served as positive patients, and the rest served as a negative control. The results are shown in Fig. 3. The AUC was 0.711 (95% CI: 0.572–0.826, *P* < 0.05), and the optimal cutoff value of DHEA was 13.28 ng/ml, with a sensitivity of 66.67% (95% CI: 30.1–92.1%), a specificity of 84.44% (95% CI: 0.5–93.5%), a positive predictive value of 46.2% (95% CI: 19.3–74.8%) and a negative predictive value of 92.7% (95% CI: 80.1–98.4%). DHEA-S at baseline had no such predictive value for the 2-day dexamethasone suppression test.

In total, 23 patients had elevated DHEA levels of more than 13.28 ng/ml in this cohort of 102 PCOS patients. According to the sensitivity of 66.67% of this index, it is predicted that 15 patients have adrenal-origin androgen dominance.

DHEA-S was used as an index representing adrenal-origin androgen excess [9–11]. In our study, patients with DHEA-S ≥ 3833.15 ng/ml (DHEA-S ≥ the 90th percentile of healthy volunteers) [12] were considered to have adrenal-origin androgen excess. In total, 14 of 51 patients underwent the dexamethasone suppression test had elevated levels of DHEA-S at baseline. In the patients of our study, a significant decrease in TT and AD following the 2-day dexamethasone administration was not parallel to adrenal-origin androgen excess. Only 1 of 9 patient who had a significant decrease in TT had elevated level of DHEA-S at baseline.

## Discussion

TT has been widely used as an evaluation index of hyperandrogenemia, but there are still some problems. First, chemiluminescence is currently used for TT determination in most clinical chemistry laboratories. However, DHEA-S which is the most abundant sex hormone precursor produced by the adrenal gland, is present in 1000-fold higher concentrations than testosterone and seriously interferes with the determination of testosterone [4]. Knudsen reported that significant differences were observed between TT measurements employing an automatic second-generation immunoassay and LC-MS/MS [13]. LC-MS/MS determination can overcome the disadvantages of chemiluminescence and is recommended for TT measurement. Second, the TT level is affected by SHBG, which is a transport carrier that binds estrogen and androgens and regulates their biological activities [14]. In our study, SHBG was lower in the PCOS patients than in healthy volunteers and was one of the factors influencing TT. Under the condition of low SHBG, if TT is used as the evaluation index, hyperandrogenemia will be underestimated. The FAI is an indicator of the TT level by SHBG adjustment and was the best index for representing hyperandrogenism in PCOS patients in our study.

AD is not as widely measured in clinical departments as TT; however, the clinical value of AD is not lower than that of TT. AD and TT, but not any other androgen precursors, are key steroid hormones in obese adolescents with PCOS [15], and AD is believed to be a sensitive biochemical marker of clinical hyperandrogenism for PCOS in type 1 diabetes mellitus [16]. O’Reilly showed that 20 in 86 patients with PCOS had elevated levels of AD but normal levels of TT and pointed out that this discrepancy may result from the effects of hyperinsulinism on SHBG binding, which does not affect AD but TT [3, 4]. Our results showed that there was no significant difference in the potency of AD or TT alone in evaluating hyperandrogenemia. However, we further found that 11 of 102 patients with PCOS had elevated levels of AD but normal levels of TT, whereas only 2 patients had elevated levels of TT but normal levels of AD. From this point of view, AD may be better than TT in assessing hyperandrogenemia. Furthermore, alternatively, AD or an FAI more than the optimal cutoff values as evidence of hyperandrogenemia significantly improved the screening efficiency.

Females have two main androgen origins: the ovary and the adrenal gland. These two glands share the same enzyme system in the process of androgen biosynthesis, and the intermediate and end products of androgen are basically the same [17]. The measurement of circulating DHEA-S is frequently used as a marker of adrenal androgen secretion [18]. Because of the high concentration of DHEA-S in plasma, the application of the chemiluminescent method in the determination of DHEA-S is relatively less affected by the other androgen precursors, but the application of LC-MS/MS can improve the accuracy and reduce the time required for the determination procedure [19–21]. PCOS patients had elevated levels of DHEA-S compared with the healthy volunteers in our study, suggesting that some PCOS patients had elevated androgens in both origination**.** The prevalence of DHEA-S excess is 10–64% in previous studies [18, 22, 23], and the prevalence was 27.4% (14/51) in our study. DHEA is a common substrate of the enzymes 3β-hydroxysteroid dehydrogenase type 2 (HSD3B2) and sulfotransferase2A1 (SULT2A1) and the relative lack of HSD3B2 expression facilitates DHEA-S synthesis [9]. This explains why DHEA is not statistically different between PCOS patients and healthy volunteers in our study. Furthermore, The C19 steroid 11-hydroxyandrostenedione (11OHA4), which is the second-most abundant steroid produced by the zona reticularis, is the precursor for the active steroids 11-ketotestosterone (11KT) and 11-ketodihydrotestosterone (11KDHT) [19]. O’Reilly and his co-workers showed that serum concentrations of 11OHA4, 11KT and 11KDHT were all significantly higher in PCOS women than controls [24]. Whether these indexes can be used as screening indicators of hyperandrogenemia in PCOS patients or to evaluate the level of adrenal androgen still needs further investigations.

Different androgen origins have different regulatory mechanisms. Ovarian androgen synthesis is regulated by LH, whereas adrenal androgen synthesis is regulated by ACTH [25]. Therefore, the dexamethasone suppression test can be used to estimate the dominant source of androgen. However, the increase in androgen from the adrenal gland and the dominance of androgen from the adrenal gland have two different meanings and are not parallel. In our study, 9 in 51 patients with PCOS had significant decreases in TT after 2-day dexamethasone administration, but only 1 of these 9 patients had elevated level of DHEA-S at baseline. Several reasons may explain the dominance of androgen from the adrenal gland in PCOS patients. First, PCOS patients had normal levels of circulating ACTH and a similar response to corticotrophin releasing hormone [5, 17], but the response of the adrenal gland to ACTH stimulation was exaggerated [17, 26, 27]. Second, previous studies suggested that testosterone may increase adrenal androgen synthesis basally and in response to ACTH [28]. Third, DHEA-S is the most abundant androgen precursor in circulation and can be transferred into testosterone in peripheral tissue. In patients with dominance of androgen from the adrenal gland, DHEA-S and DHEA synthesized by adrenal gland may be used as precursors for ovarian synthesis of AD and testosterone.

The most important implication of determining the dominant source of androgen for PCOS patients is to assist in making the treatment plan. Vanky showed that six-month, low-dose dexamethasone treatment further reduced androgen levels in metformin-treated PCOS women [29]. Physiological doses of dexamethasone or prednisone can directly lower adrenal androgen output and are recommended by related guidelines [30], and this kind of medicine may be useful, especially for PCOS patients with adrenal-origin androgen dominance.

## Conclusion

AD measured by LC-MS/MS can represent hyperandrogenemia in PCOS patients and, combined with TT or the FAI, can improve the screening efficiency of hyperandrogenemia. Seventeen percent of patients had adrenal-origin androgen dominance, with TT significantly decreasing after 2 days of dexamethasone administration. A total of 27% of patients had DHEA-S excess, but DHEA-S excess was not parallel to adrenal-original androgen dominance in the patients of this study.

## Data Availability

The datasets used and/or analyzed during the current study are available from the corresponding author on reasonable request.

## Abbreviations

11KDHT: 11-ketodihydrotestosterone
11KT: 11-ketotestosterone
11OHA4: 11-hydroxyandrostenedione
ACTH: Adrenocorticotropin
AD: Androstenedione
AUC: Area under the curve
DHEA: Dehydroepiandrosterone
DHEA-S: Dehydroepiandrosterone sulfate
FAI: Free androgen index
FSH: Follicle-stimulating hormone
HSD3B2: 3β-hydroxysteroid dehydrogenase type 2
LC-MS/MS: Liquid chromatography-mass spectrometry
LH: Luteinizing hormone
PCOS: Polycystic ovary syndrome
ROC: Receiver operating characteristics
SHBG: Sex hormone binding globulin
SULT2A1: Sulfotransferase2A1
TT: Total testosterone
